# Supplementary material for: Impact of driving characteristic parameters and vehicle type on fuel consumption and emissions performance over real driving cycles
Source: PLoS One. 2025 Jan 13;20(1):e0317098. doi: 10.1371/journal.pone.0317098 (PMC11729959; doi:10.1371/journal.pone.0317098)
Supplement: Attachment — Submitted filename: Authors Response-PLOS One_v4.docx [file pone.0317098.s001.docx]

**Dear Editor-in-Chief,**

PLOS ONE

Authors thank you for giving me the opportunity to revise our paper titled “Impact of driving characteristic parameters and vehicle type on fuel consumption and emissions performance over real driving cycles”. We appreciate the valuable feedback and comments provided by the reviewers, which have helped us to improve the quality and clarity of the manuscript. In this revision letter, we have considered each of the reviewers' comments and explained how we have addressed them in the revised version of the paper. We hope that the yellow-colored highlighted changes we have made will sufficiently address the concerns raised and that the revised manuscript will be suitable for publication in PLOS ONE.

Regards,

**M. Azadi, PhD.**

Faculty of Mechanical Engineering, Semnan University, Semnan, Iran

**Journal requirements:**

1) We've checked your submission and before we can proceed, we need you to address the following issues:

Please note that PLOS utilizes the CC BY 4.0 license (<https://creativecommons.org/licenses/by/4.0/>) which means that all material on our website is freely available online, and any third party is permitted to access, download, copy, distribute, and use these materials in any way, even commercially, with proper attribution. If the figures have already been published, authors must provide proper attribution, referencing the source clearly, and obtain permissions if the image is copyrighted.
Before we can proceed with your manuscript, we ask that you please clarify the following:
1) Please explain the specific sources where the authors obtained the images in Figure 1 and the map in Figure 2 in your submission or if the authors created the images and map themselves.
2) Please state whether the images in Figure 1 have been previously copyrighted.
3) If any of the images in Figure 1 have been previously copyrighted, we require specific consent from the copyright holder to publish these images in PLOS ONE, under the CC BY 4.0 license. To seek permission from the copyright owner to publish these figures under the Creative Commons Attribution License (CCAL), CC BY 4.0, please contact them with the following text and PLOS ONE Request for Permission form (<https://journals.plos.org/plosone/s/file?id=c4ff/Content%20Copyright%20Permission%20Form.pdf>):
“I request permission for the open-access journal PLOS ONE to publish XXX under the Creative Commons Attribution License (CCAL) CC BY 4.0 (<http://creativecommons.org/licenses/by/4.0/>). Please be aware that this license allows unrestricted use and distribution, even commercially, by third parties. Please reply and provide explicit written permission to publish XXX under a CC BY license.”
Please upload the granted permission to the manuscript as another file. In the figure caption of the copyrighted figure, please include the following text: “Republished from [ref] under a CC BY license, with permission from [name of publisher], original copyright [original copyright year].”
Please note that RightsLink permission forms often impose use restrictions that are incompatible with our CC BY 4.0 license, and we are therefore unable to accept these permissions. For this reason, we strongly recommend contacting copyright holders with the PLOS ONE Request for Permission form.
If you are unable to obtain permission from the original copyright holder, please either remove the figure or supply a replacement figure that complies with the CC BY 4.0 license. Please check copyright information on all replacement figures and update the figure caption with source information.
**Author’s Response:** *Thank you for bringing this to our attention. We have revised Figure 1 to remove the previous image, replacing it with a new illustration that does not contain any copyrighted material. Additionally, we have deleted Figure 2 and replaced it with a new figure that is an original creation of the authors and does not require any copyright permissions.*

*We have ensured that all figures and illustrations used in our revised submission are original or use public domain images, and we have taken all necessary steps to ensure their proper attribution. We appreciate your diligence in helping us maintain the integrity and validity of our research, and we believe that these changes address your concerns regarding copyright and image usage.*

**Fig. 1.** The steps of the research as a flowchart

**Fig. 2.** Representative of sample routes 1 and 3. Figure created by the authors using Paint.NET, Adobe Photoshop, Microsoft Visio Pro and Autodesk SketchBook.

2) We note that you state the following regarding your Figures: "Thank you for bringing this to our attention. We have revised Figure 1 to remove the previous image, replacing it with a new illustration that does not contain any copyrighted material. Additionally, we have deleted Figure 2 and replaced it with a new figure that is an original creation of the authors and does not require any copyright permissions.

We have ensured that all figures and illustrations used in our revised submission are original or use public domain images, and we have taken all necessary steps to ensure their proper attribution. We appreciate your diligence in helping us maintain the integrity and validity of our research, and we believe that these changes address your concerns regarding copyright and image usage."
At this time, please clarify what, if any, software was used to create the map for Figure 2 and if any 3rd party data was used.

**Author’s Response:** *Thank you for your attention. In this study, the maps in Figure 2 were created with the help of professional software, including Paint.NET version: 5.0.13, Adobe Photoshop version: 22.5.7 and Autodesk SketchBook version: 8.8.36. In addition, the labeling of areas and the layout of sample routes have also been done with the help of Microsoft Visio Pro and PowerPoint 2019. We assure you that no third-party data has been used in this way, and all material and intellectual rights belong to Plus One Journal. The Transformations are added to the description of figure 2 as shown in the following.*

**Fig. 2.** Representative of sample routes 1 and 3. Figure created by the authors using Paint.NET, Adobe Photoshop, Microsoft Visio Pro and Autodesk SketchBook.

3) When submitting your revision, we need you to address these additional requirements. Please ensure that your manuscript meets PLOS ONE's style requirements, including those for file naming. The PLOS ONE style templates can be found at https://journals.plos.org/plosone/s/file?id=wjVg/PLOSOne_formatting_sample_main_body.pdf and <https://journals.plos.org/plosone/s/file?id=ba62/PLOSOne_formatting_sample_title_authors_affiliations.pdf>

**Author’s Response:** *Thank you for pointing out the additional requirements. We will ensure that my revised manuscript meets PLOS ONE's style requirements.*

4) You indicated that ethical approval was not necessary for your study, although the study appears to report data collected about a human participant while driving. We understand that the framework for ethical oversight requirements for studies of this type may differ depending on the setting and we would appreciate some further clarification regarding your research. Could you please provide further details on why your study is exempt from the need for approval and confirmation from your institutional review board or research ethics committee (e.g., in the form of a letter or email correspondence) that ethics review was not necessary for this study? Please include a copy of the correspondence as an ""Other"" file.

**Author’s Response:** *Thank you for your comment. We understand your concerns regarding the ethical approval of our study, and we appreciate your request for clarification.*

*This study only contains data of global navigation satellite systems, such as GPS devises, installed in five different vehicles. In order to eliminate personal interests in the collected data, all vehicles were driven by the corresponding author of this paper (i.e. Dr. Mohammad Azadi). He is aware of the objectives of the study and has a lot of experience in similar works. Therefore, the current pilot study is exempt from a specific ethical approval. The following statement is added at the end of the current manuscript.*

**Ethical approval** **statement**

The reported data in this study only involves recording data from the vehicles driven by the corresponding author, who was aware of the study's objectives. Therefore, the current pilot study is exempt from a specific ethical approval.

5) Please note that PLOS ONE has specific guidelines on code sharing for submissions in which author-generated code underpins the findings in the manuscript. In these cases, all author-generated code must be made available without restrictions upon publication of the work. Please review our guidelines at https://journals.plos.org/plosone/s/materials-and-software-sharing#loc-sharing-code and ensure that your code is shared in a way that follows best practice and facilitates reproducibility and reuse.

**Author’s Response:** *Thanks for your comment, I will make sure to comply with PLOS ONE's guidelines on code sharing and provide all author-generated code without restrictions upon publication of the work.* *All data related to this study can be found at:* [*https://github.com/elmirabagheri/Driving_Characteristic_and_Development*](https://github.com/elmirabagheri/Driving_Characteristic_and_Development)

**Data availability**

All Code and data related to this study are publicly available at:

<https://github.com/elmirabagheri/Driving_Characteristic_and_Development>.

6) We note that Figure 2 in your submission contain map images which may be copyrighted. All PLOS content is published under the Creative Commons Attribution License (CC BY 4.0), which means that the manuscript, images, and Supporting Information files will be freely available online, and any third party is permitted to access, download, copy, distribute, and use these materials in any way, even commercially, with proper attribution. For these reasons, we cannot publish previously copyrighted maps or satellite images created using proprietary data, such as Google software (Google Maps, Street View, and Earth). For more information, see our copyright guidelines: <http://journals.plos.org/plosone/s/licenses-and-copyright>. We require you to either present written permission from the copyright holder to publish these figures specifically under the CC BY 4.0 license, or remove the figures from your submission: a. You may seek permission from the original copyright holder of Figure 2 to publish the content specifically under the CC BY 4.0 license. We recommend that you contact the original copyright holder with the Content Permission Form (http://journals.plos.org/plosone/s/file?id=7c09/content-permission-form.pdf) and the following text: “I request permission for the open-access journal PLOS ONE to publish XXX under the Creative Commons Attribution License (CCAL) CC BY 4.0 (http://creativecommons.org/licenses/by/4.0/). Please be aware that this license allows unrestricted use and distribution, even commercially, by third parties. Please reply and provide explicit written permission to publish XXX under a CC BY license and complete the attached form.” Please upload the completed Content Permission Form or other proof of granted permissions as an "Other" file with your submission. In the figure caption of the copyrighted figure, please include the following text: “Reprinted from [ref] under a CC BY license, with permission from [name of publisher], original copyright [original copyright year].” b. If you are unable to obtain permission from the original copyright holder to publish these figures under the CC BY 4.0 license or if the copyright holder’s requirements are incompatible with the CC BY 4.0 license, please either i) remove the figure or ii) supply a replacement figure that complies with the CC BY 4.0 license. Please check copyright information on all replacement figures and update the figure caption with source information. If applicable, please specify in the figure caption text when a figure is similar but not identical to the original image and is therefore for illustrative purposes only. The following resources for replacing copyrighted map figures may be helpful: USGS National Map Viewer (public domain): <http://viewer.nationalmap.gov/viewer/> The Gateway to Astronaut Photography of Earth (public domain): <http://eol.jsc.nasa.gov/sseop/clickmap/> Maps at the CIA (public domain): https://www.cia.gov/library/publications/the-world-factbook/index.html and <https://www.cia.gov/library/publications/cia-maps-publications/index.html> NASA Earth Observatory (public domain): <http://earthobservatory.nasa.gov/> Landsat: <http://landsat.visibleearth.nasa.gov/> USGS EROS (Earth Resources Observatory and Science (EROS) Center) (public domain): [http://eros.usgs.gov/#](http://eros.usgs.gov/) Natural Earth (public domain): <http://www.naturalearthdata.com/>

**Author’s Response:** *We appreciate your diligence in bringing this to our attention. To address your concern, we have removed figure 2 with a new figure that is an original creation of the authors and does not require any copyright permissions.*

**Fig. 2.** Representative of sample routes 1 and 3. Figure created by the authors using Paint.NET, Adobe Photoshop, Microsoft Visio Pro and Autodesk SketchBook.

**Additional Editor Comments:**

Dear Author,

After the reviews conducted by two independent experts on the proposed topic, my recommendation is to address all the concerns that remain outstanding according to one of the reviewers (major revisions/rejection). Once these concerns have been addressed satisfactorily, a new round of reviews will be conducted, involving a third reviewer.

One aspect I have observed is that the authors do not include a research methodology that is highly relevant for this type of study on current driving performance: naturalistic driving experiments. Among other studies, the authors may consider evaluating research related to the Extraction of naturalistic driving patterns or the Representation of kinematic driving behavior using Geographic Information Systems, where driving patterns of individuals under real conditions are precisely addressed and visualized.

**Author’s Response:** *Thank you for your comment. We are pleased to address your concerns regarding the methodology employed in this study. We have added a detailed section on our methodology, including a representation of the steps outlined in Fig. 1.*

*In this section, we explain the methodology employed in our study, including the calculation of sample routes, selection of test vehicles, and processing of naturalistic data to develop driving cycles. We also detail the parameters used to evaluate driving behavior, including the reduction of these parameters to two sensitive parameters using Principal Component Analysis (PCA). Additionally, we describe the use of the K-means algorithm to construct driving cycles and the simulation of vehicles in MATLAB/Simulink using input driving cycles.*

*We believe that our revised methodology section provides a clear and comprehensive explanation of the methods used in our study, and addresses the concerns raised by the reviewer regarding the lack of detail on our methodology.*

In this section, the methodology employed in this study is explained in detail, with a representation of the steps outlined in Fig. 1. The subsequent explanation is also provided.

Firstly, the sample routes are calculated by considering traffic data from various types of roads and populous areas in Tehran city. Then, vehicles are selected based on their specifications, such as powertrain, engine types, and body styles, and choose the most popular ones. This ensures that the test vehicles are representative of real driving conditions. To control for individual driving behavior and focus on the impact of vehicle type on driving performance, a single driver drove all sample routes. This allows to isolate the effect of vehicle characteristics on the results. The naturalistic data are then processed to develop driving cycles, which are crucial for understanding the behavior of vehicles under real-world conditions. The 12 driving characteristic parameters calculated are indeed related to kinematic driving behavior, and these parameters are used to identify the most influential factors on the results. By reducing these parameters to two sensitive parameters using PCA, the underlying dynamics of vehicle behavior can better understand.

The K-means algorithm is employed to construct driving cycles for five vehicles in all sample routes. After that, vehicles are simulated in MATLAB/Simulink according to their specifications, and input the related driving cycles as scenarios to each vehicle. By running simulations, fuel consumption and emissions are obtained for each vehicle. This allows to examine the impact of vehicle technologies on fuel consumption and emissions. After analyzing the driving characteristic parameters of each driving cycle, the impact of vehicle technologies along with their corresponding fuel consumption and emissions data, to see how each vehicle affects these parameters.

**Fig. 1.** The steps of the research as a flowchart

**Reviewer #1:**

The manuscript proposes "Impact of driving characteristic parameters and vehicle type on fuel consumption and emissions performance over real driving cycles."

The reviewer's concerns are as follows:

1) The novelty of this study is not clear. To enable readers to follow the article more quickly and effectively, a flowchart summarizing the article should be added to the introduction section.

**Author’s Response:** *First of all, we should thank the respected reviewers for reviewing the manuscript. We have tried our bests to address all comments in the revised articles with yellow-colored highlights for changes.*

*In order to address this comment, we have revised our previous flowchart of methodology to a complete one, which now includes all the steps involved in our study. We have also added an overview of methodology in this section, providing a detailed explanation of the research design, data collection, and data analysis procedures used in our study.*

***Additionally, we have revised the novelty of the study in a bullet point at the end of the introduction, highlighting the main contributions and advancements of our research.***

The innovations of this paper can be summarized as follows:

- Development of real driving cycles using K-means clustering and PCA, combined with real driving data from four distinct routes and one driver to capture representative driving patterns.
- Utilization of a simulation-based approach with MATLAB Powertrain Blockset and Simulink Design to simulate reference vehicles and generate fuel consumption and emissions data for real driving cycles, as well as two standard driving cycles.
- Comparative analysis is conducted to examine the impact of different powertrains, engines, and body styles on driving characteristic parameters, exhaust emission behavior, and fuel consumption of conventional vehicles and a hybrid vehicle.

**2. Methodology**

In this section, the methodology employed in this study is explained in detail, with a representation of the steps outlined in Fig. 1. The subsequent explanation is also provided.

Firstly, the sample routes are calculated by considering traffic data from various types of roads and populous areas in Tehran city. Then, vehicles are selected based on their specifications, such as powertrain, engine types, and body styles, and choose the most popular ones. This ensures that the test vehicles are representative of real driving conditions. To control for individual driving behavior and focus on the impact of vehicle type on driving performance, a single driver drove all sample routes. This allows to isolate the effect of vehicle characteristics on the results. The naturalistic data are then processed to develop driving cycles, which are crucial for understanding the behavior of vehicles under real-world conditions. The 12 driving characteristic parameters calculated are indeed related to kinematic driving behavior, and these parameters are used to identify the most influential factors on the results. By reducing these parameters to two sensitive parameters using PCA, the underlying dynamics of vehicle behavior can better understand.

The K-means algorithm is employed to construct driving cycles for five vehicles in all sample routes. After that, vehicles are simulated in MATLAB/Simulink according to their specifications, and input the related driving cycles as scenarios to each vehicle. By running simulations, fuel consumption and emissions are obtained for each vehicle. This allows to examine the impact of vehicle technologies on fuel consumption and emissions. After analyzing the driving characteristic parameters of each driving cycle, the impact of vehicle technologies along with their corresponding fuel consumption and emissions data, to see how each vehicle affects these parameters.

**Fig. 1.** The steps of the research as a flowchart

2) Authors have not provided comparison between their proposed methodology and the previous ones in the introduction. According to the current introduction, there is no contribution in the current study.

**Author’s Response:** *Thank you for your insightful comment. In order to address your comment, we have added the methodology of previous research that I didn’t mention. Also, some sentences are added to compare our proposed methodology with previous ones in introduction section, as you had suggested.*

Chong et al. [9], analyzed vehicles using a Portable Emissions Measurement System (PEMS) that included a Global Positioning System (GPS). They found that higher levels of NOx emissions were associated with increased speeds, stronger accelerations, and reduced exhaust gas recirculation. During engine conversions, excessive emissions of HC and CO were frequently observed, particularly during acceleration. Sofwan and Latif [10], found that operating vehicles at low speeds, which improves fuel economy, leads to reduced emissions of CO and NOx. The results were obtained through on-board emissions testing and were conducted on main streets in the heart of Kuala Lumpur's urban environment. However, this operating condition is associated with increased emissions of CO_2_. Relying solely on average speed does not adequately capture driving behavior.

The wide range of vehicle types and their distinct characteristics make it impractical to adopt a single universal driving cycle for all of them, which can result in either underestimating or overestimating emission factors. In contrast to previous studies that relied on standardized drive cycles developed in America and Europe or real data of fuel consumption and emissions, our study creates real driving cycles that are specifically tailored to the unique driving patterns of emerging automobile markets like Iran. This is achieved by collecting data along four different route types and using a combination of Principal Component Analysis (PCA) and K-means clustering to develop driving cycles that accurately represent the driving patterns of these regions.

3) It is recommended to add the following reference. a) Adak, S., Cangi, H., Kaya, R., Yılmaz, A. S. (2022). Effects of Electric Vehicles and Charging Stations on Microgrid Power Quality. Gazi University Journal of Science Part A: Engineering and Innovation, 9(3), 276-286. <https://doi.org/10.54287/gujsa.1153313>

**Author’s Response:** *Thanks for your valuable suggestion. The mentioned reference is added to the revised paper in Introduction section. Also, other references are added to make the paper more comprehensive in result and discussion section.*

**Reviewer #2:**

1) This paper intended to consider the driving characteristic parameters, fuel consumption, and emissions performance of vehicles with different technologies such as powertrain, engine, and body style under both real and standard drive cycles. 19 drive cycles were created by utilizing the K-means and PCA algorithms, based on the selection of four sample routes. The vehicles underwent modeling in MATLAB/Simulink based on their specifications, and real and standard drive cycles were simulated to measure their fuel consumption and emissions. Vehicles #1, 2, 3, and 4 are conventional vehicles and vehicle #5 is a hybrid vehicle considered. The way to choose vehicles considered different types of powertrains, body styles, and the presence of turbocharged. For driving characteristic parameters, emissions, and fuel consumption comparisons, three vehicle models for three powertrains (gasoline, hybrid electric, and dual fuel), two types of engines (turbocharged and naturally aspirated), and two vehicle body styles (i.e., sedan, SUV), are used. To reduce the influence of the driver's behavior on the test results, the same driver drove all routes.

Even the driving cycles for 5 vehicles on the same route were very different from each other. (Fig.3 and Fig.5). This will negatively affect the analysis to be performed. No explanation was given for this.

**Author’s Response:** ***Thank you for the comment.*** *We understand your concern about the driving cycles. However, we would like to provide some additional context to alleviate concerns about the driving cycles. Although we acknowledge that the speed ranges of the driving cycles for each vehicle on the same routes are similar, we understand that the variability in data collection timing and dates may result in differences in driving cycles for the same routes. To mitigate this issue, we repeated the same procedure for both Federal Test Procedure 75 (FTP-75) and Urban Dynamometer Driving Schedule (UDDS) cycles with the same vehicle category. This allowed us to eliminate the influence of real-world driving conditions and provide a more controlled comparison between different vehicles. The changes are provided in Result and discussion section as follow:*

In this part, the driving cycles developed through K-means clustering and PCA algorithm are utilized to estimate the driving characteristic parameters, fuel consumption, and emissions of vehicles. Initially, the 12 driving characteristic parameters used in the PCA algorithm are calculated for the final driving cycles. Subsequently, by simulating the vehicles and inputting the driving cycles, fuel consumption and emissions are calculated. Finally, each vehicle is compared with other vehicles in terms of powertrain, engine downsizing, and body style based on their characteristics. To eliminate the influence of real-world driving conditions, the same procedure is repeated for the Federal Test Procedure 75 (FTP-75) and the Urban Dynamometer Driving Schedule (UDDS) cycles, using the same vehicle category [25, 26].

2) Vehicle simulation steps performed in the MATLAB/Simulink program are not detailed enough. How are fuel consumption and emissions calculated? What is the difference between the calculations for a hybrid vehicle (Toyota Prius) and a dual fuel vehicle and the calculations for a conventional vehicle?

**Author’s Response:** *I appreciate your feedback on the vehicle simulation steps performed in the MATLAB/Simulink program. We provide more details on the calculations used to estimate fuel consumption and emissions of all vehicles in Vehicle simulation section.*

In this section, vehicles are simulated by using MATLAB/Simulink with the Powertrain Blockset, which is a powerful tool for data-driven modeling and analyzing the performance of vehicles [24]. The Powertrain Blockset provides a comprehensive set of blocks and components that allow to simulation and design of powertrains, including engines, transmissions, drivelines, and other vehicle subsystems. The realistic model of all five vehicles was simulated separately in this environment, each of which consists of six main blocks. The names and connection order of these blocks are shown in Fig. 6.

**Fig. 6.** Steps and structure of designing real vehicle models with MATLAB Virtual Vehicle Composer

Scenarios block is the starting point of modeling, where the behavior of the driver and their maneuvers are modeled in the form of drive cycles. The output of this block serves as input ports for other subsequent blocks. Within the Scenarios block, there are two constant inputs for driver command and driver reference, as well as one feedback input for triggering subsystems. These inputs are defined as 1-D arrays. The concepts of vehicle dynamics are calculated based on coordinate systems, which are defined in the Z-down and Z-up orientations with the sub-blocks of SAE J670 and ISO 8855, respectively. The parameters of this block are independent of the technical specifications of the vehicle, meaning that the initial values of lateral position and vertical position can be set uniformly for all five vehicles. In this case, the initial values were set to 5.7 and 0, respectively.

The driver's behavior and vehicle control are affected by the surrounding environment and road conditions. In order to include these factors, a block named Environment is designed at the beginning of the system and runs parallel to the Scenarios block. The Environment block has a common input port with the scenarios block and its output is connected to other blocks. The parameters of this block include ambient temperature, ambient atmospheric pressure and wind velocity. The wind velocity values in the global inertial frame are represented by three separate ports in X, Y, and Z coordinates, which are connected to each other through an embedded subsystem. In accordance with the detailed analysis of the model, road friction is designed as a subsystem comprising three levels: constant friction, varying friction and three-axle constant friction. These levels are based on wheel parameters, body aerodynamics, suspension system and chassis. The initial values for ambient temperature, ambient atmospheric pressure, and wind velocity for all vehicles are set to 298.15 K (25 °C), 101325 pascal, and 0 m/s, respectively. This provides a consistent set of conditions for a fair comparison of fuel consumption and emissions across all vehicles in this study.

The Driver Commands block can simulate the vehicle's behavior in one of four modes: longitudinal driver, predictive driver, predictive Stanley driver and no driver. In each of these modes, the vehicle reference, vehicle feedback, steering command, gear command, brake command, acceleration command, and environment ports serve as inputs. The primary function of this block is to produce acceleration, braking, gear, and steering signals in various ways. For example, in the longitudinal driver mode, the block generates normalized acceleration and braking commands (between 0 and 1) to track the longitudinal drive cycle by applying a speed-tracking controller. More details of simulating the Driver Command block in Simulink are shown in Fig 7.

**Fig. 7.** Simulation of the Driver Commands block in longitudinal driver mode

In this section, the Driver Commands block with the longitudinal driver mode is used to model the dynamic response of the driver in all vehicles. Additionally, for each signal, three external actions (hold, override and disable) are defined, which are applied to closed-loop commands with a default priority. For each vehicle, three types of rear, neutral and drive shifts (i.e. R, N, D gears) are included with a change time of 0.1 seconds and the velocity gain breakpoints are set between 0 and 100. The details and other parameter values for this block are shown in Table 3 for each vehicle.

The Controller block is a main component in the simulation, connected bilaterally with the Vehicle block. This block is comprised of seven subsystems named Vehicle Control Unit (VCU), Engine Control Unit (ECU), Transmission Control Unit (TCU), Brake Control Unit (BCU), Active Differential Control (ACD), Battery Management System (BMS), and Thermal Management (TM). VCU is a master-slave architecture designed to control the powertrain in vehicles. It is integrated into the engine controller ECU, which is capable of estimating the parameters of open-loop air, fuel, spark, and cam-phaser actuator from engine speed and torque signals. Gear shifting is predicted in TCU, which is determined from the inputs of deceleration, acceleration, engine speed, and instantaneous vehicle speed.

**Table 3.** Parameter values of Driver Commands block

| *Vehicle* | | | | | *Parameters* |
| --- | --- | --- | --- | --- | --- |
| *#5* | ***#4*** | ***#3*** | ***#2*** | ***#1*** |  |
| 1545 | 1315 | 1700 | 1414 | 1410 | ***Mass*** |
| 0.1 | 0.1 | 0.1 | 0.1 | 0.1 | ***Driver Response Time*** |
| 4.1 | 4.1 | 4.1 | 4.1 | 4.1 | ***Preview Distance*** |
| 2.5 | 2.5 | 2.5 | 2.5 | 2.5 | ***Rolling Resistance Coe.*** |
| 0.30 | 0.30 | 0.45 | 0.30 | 0.25 | ***Aerodynamic Drag Coe.*** |
| 9.81 | 9.81 | 9.81 | 9.81 | 9.81 | ***Gravitational Constant*** |
| 0 | 0 | 0 | 0 | 0 | ***Initial Gear*** |
| 0.12 | 0.12 | 0.10 | 0.12 | 0.11 | ***Gear Shift Delay*** |

The Vehicle block is the most complex component in simulating the real-vehicle model, as it determines the performance of various parts of the vehicle, including the engine, drivetrain, body, suspension system, chassis, vehicle electronics, sensors, and pedal cluster. The engine subsystem is responsible for modeling the engine in terms of maximum torque, fuel flow, engine efficiency factor and engine speed. For the test vehicles in this study, the engine configuration was selected as Mapped Spark Ignition (SI), with the exception that vehicles #2 and #3 also feature turbochargers. The engine subsystem's basic inputs include engine speed, environment, vehicle body, and engine command. For each vehicle, a maximum torque curve is mapped to the model, which determines the maximum brake torque in terms of engine speed. The average brake specific fuel consumption and fuel specific gravity values are assumed to be equal to 350 g/kwh and 0.754, respectively. Other effective configurations in this subsystem include the number of cylinders, gas constant air, and air standard temperature, which are selected according to the characteristics of each vehicle. To simplify the analysis of results, the parameters of air mass flow, exhaust temperature, actual brake torque, and fuel mass flow in terms of engine speed and commanded torque are considered the same for all vehicles. The values of these parameters are shown in Fig. 8.

a)	b)

c)	d)

**Fig. 8.** Displaying the parameters of a) air mass flow map, b) exhaust temperature map, c) actual brake torque map, and d) fuel mass flow map according to engine speed and commanded torque

The drivetrain subsystem consists of two parts: drivetrain layout and actuators, which are designed to model the powertrain and transmission of vehicles. The vehicle electronics subsystem is responsible for controlling the voltage and current of fuel cells. However, the presence of a HEV in this study has resulted in a significant difference between vehicle #5 and the other four conventional vehicles. This distinction is made up of the battery system and electric machines to supply the vehicle's energy according to Fig. 9. The parameters specific to this vehicle are as follows: the number of cells in series is 96, the initial battery capacity is 3.18 Ampere-hours (Ah) and the rated capacity at nominal temperature is 5.3 Ah.

**Fig. 9.** Simulation of the vehicle electronics subsystem for vehicle #5

Finally, the output of the Vehicle block is fed back into the Scenarios and Environment blocks to ensure the stability of the system. After completing the model design, different drive cycles are applied in the Scenarios block, and then the model is run. The outputs of each port can be visualized using scopes in the Visualization block. It should be noted that the calculation of fuel consumption and emissions in this section is obtained through calibration maps in the Mapped SI Engine block. These maps model engine behavior and control parameters, which are defined as separate lookup table for each type of pollution (i.e., CO, HC, NOx, and CO_2_). To be more precise, engine-out emissions and engine fuel flow obtained for each vehicle are a function of engine torque and speed, illustrating the engine's response to control inputs in different situations.”

3) The result, which includes the effect of driving characteristic parameters, powertrain type, engine downsizing and body style on fuel consumption and emissions, was not found scientifically sufficient.

**Author’s Response:** *Thank you for your comment. We have taken your suggestion into consideration and have revised the results and discussion section to improve the scientific sufficiency of our findings. In response to your comment, we have removed the explicit sentences that were previously included in the results section and instead presented our findings in a more nuanced and scientific manner. We have also added relevant references to support our conclusions and provide context for the results. Additionally, the conclusion is revised according to the results.*

*In “****Effect of driving characteristic parameters on fuel consumption and emissions****” Section:*

Driving behavior, traffic conditions, vehicle technology, and sample routes all play a significant role in determining the driving characteristic parameters of a vehicle, as well as its fuel consumption and emissions. Each vehicle has its unique driving cycle, which leads to varying values of these parameters. Table 4 demonstrates the driving characteristic parameters and Table 5 illustrates fuel consumption and emissions for vehicles on the sample routes. Fuel consumption, CO_2_, and other emission rates are estimated in l/100km, g/km, and g/mi, respectively. The sample routes are identified by R1, R2, R3, and R4, which correspond to routes 1, 2, 3, and 4, respectively.

As mentioned earlier, fuel consumption and emissions are influenced by a multitude of factors, making it a challengeable to account for all of them. However, several key variables have been identified as significant contributors to increased fuel consumption and emissions. These include traffic congestion, which can lead to prolonged idling times and reduced engine efficiency, as well as aggressive driving behaviors such as excessive speed and acceleration. Furthermore, the vehicle's technological features, including its engine management system and emission control devices, also play a crucial role in determining fuel consumption and emissions. To illustrate the effects of these parameters on fuel consumption and emissions, the driving behavior of five vehicles on four sample routes were examined.

The results indicate that vehicles with lower traffic congestion (e.g., shorter idling times) exhibit lower fuel consumption and emissions compared to those with higher traffic congestion (e.g., longer idling times). For instance, vehicle #1 on sample route 4 experienced a lower period (0.83%) of idling time, resulting in lower fuel consumption and emissions compared to other sample routes.

The experimental findings also revealed that extended idling consumes more fuel compared to restarting, but restarting the engine leads to significantly increased emissions of CO, HC, and NOx relative to a brief period of idling [27]. These findings suggest higher traffic congestion, resulting in increased fuel consumption and emissions compared to other sample routes. However, the extended idling time is not always providing more fuel consumption and emissions. For example, vehicle #4 on sample route 1 recorded the highest idle time percentage of approximately 15.59%. The prolonged idle time may have been caused by waiting at a traffic light, resulting in reduced fuel consumption and subsequently lower emission levels. The vehicle's low acceleration and deceleration, as well as its average driving speed of around 43.92 km/h, may have contributed to this prolonged idle time.

The results also show that turbocharged vehicle #2 on sample route 4, with lowest idle time percentage (1%) and highest acceleration rate (44.03%), and an average driving speed of 49.97 km/h, exhibited increased emissions of CO and HC, while fuel consumption and CO_2_ emissions remained relatively low, likely due to the activation of turbocharging at high speeds. For this turbocharged vehicle, an average driving speed tend to exhibit higher emissions of CO and HC, whereas those with lower average driving speeds tend to exhibit lower emissions [14]. Similarly, vehicle #3, a turbocharged SUV, followed this trend. During sample route 1, vehicle #3 had a shorter driving time (around 87.23%) with a lower average driving speed (35.03 km/h) and acceleration (37.03%), which was likely due to heavy traffic conditions leading to increased fuel consumption and CO_2_ emissions [28].

By examine other driving characteristic parameters, it is evident that the standard deviation of speed and acceleration, as well as idle time, are critical variables that exhibit significant correlations with fuel consumption and emissions. Specifically, the findings suggest that increased idle time, a higher standard deviation of acceleration, and a decreased standard deviation of acceleration are associated with higher fuel consumption and CO_2_ emissions in conventional vehicles. Additionally, a higher standard deviation of speed is linked to lower CO and HC emissions in conventional vehicles, whereas this trend is reversed for turbocharged vehicles.

Vehicle #5 stands out from other vehicles due to its ability to reduce fuel consumption while idling [29]. On sample route 3, the vehicle exhibited longer idle times and shorter periods of constant speed. Its average driving speed was lower, which resulted in decreased fuel consumption, CO, and HC emissions, but increased CO_2_ emissions compared to other vehicles.

*In “****Effect of powertrain type on fuel consumption and emissions****” Section:*

To investigate the impact of powertrain type on fuel consumption and emissions, a comparative study was conducted on three vehicles: a gasoline vehicle (vehicle #1), a hybrid vehicle (vehicle #5), and a dual fuel vehicle (vehicle #4). The results of the comparison are presented in the form of a Box & Whisker diagram in Fig. 10.

Fig. 10 displays the distribution of fuel consumption and emissions for each vehicle type, with the median value represented by the middle line of each box. The interquartile range (IQR) is defined as the interval between the first quartile (Q1) and the third quartile (Q3), which demarcates the boundaries of the box. The height of the box indicates the dispersion of the data, with higher values indicating a wider range. The comparison was conducted using standard driving cycles, presented in Table 6.

As shown in Fig. 10 and Table 6, the hybrid vehicle exhibits significant advantages in terms of fuel consumption compared to gasoline (about 39%) and dual fuel vehicles (about 26%) under both standard and real driving cycles. This is attributed to the frequent utilization of the braking system or deceleration, which enables the capture and conversion of available energy [31]. The CO_2_ emissions of the hybrid vehicle demonstrate a significant decrease of approximately 34% compared to vehicle #1 and 10% compared to vehicle #4, primarily due to engine shutdown during idle periods and the frequent utilization of electric energy sources in place of the engine's power [31].

The unique characteristics of the two subsystems in the hybrid vehicle, including brushless electric motors/generators and planetary gearboxes, played a key role in analyzing the results and generalizing them to other HEVs. In addition, the idle-off feature in this vehicle takes advantage of opportunities to turn off the fueling system and reduce emissions when idling in high-traffic density cycles, which is a significant advantage over other vehicles that idle their engines, leading to increased greenhouse gas production. The technology used in power transmission gearboxes also had a significant impact on energy consumption and emissions. Conventional vehicles with automatic or manual gearboxes are limited by their gear ratios, which can lead to inefficient engine operation and reduced energy production. In contrast, hybrid vehicles with planetary gearboxes have unlimited gear ratios available, allowing for optimal engine operation and reduced fuel consumption.

A comparison between gasoline and dual fuel vehicles reveals that vehicle #4, a dual fuel vehicle, exhibited a significant 18% improvement in fuel efficiency compared to vehicles running solely on gasoline. The combustion of Compressed Natural Gas (CNG) in dual fuel vehicles is characterized by a lower carbon content compared to gasoline, which results in decreased fuel consumption. Additionally, vehicle #4 demonstrated lower CO_2_ emissions, by approximately 28%, compared to vehicle #1. It is attributed to the combustion of CNG, which is characterized by a lower carbon content compared to gasoline, resulting in decreased CO_2_ emissions and potentially lower fuel consumption [32, 33].

In terms of CO and HC emissions, the hybrid vehicle emitted higher levels of CO compared to gasoline and dual fuel vehicles, primarily due to the increased power demands placed on internal combustion engines in hybrid electric vehicles, which are necessitated by the simultaneous powering of both wheels and batteries. This may also be attributed to the shorter running time and longer intervals between engine cycles may, which may result in inadequate engine warm-up, thereby contributing to increased CO emissions [11]. In contrast, HC emissions were lower in the hybrid vehicle compared to gasoline and dual fuel vehicles under standard drive cycles, but not under real drive cycles. This is likely due to more aggressive driving conditions in real driving conditions, which can lead to incomplete combustion of fuel. It is also noteworthy that the CO and HC emissions of gasoline vehicle tend to be higher when than dual fuel vehicle by approximately 45% and 39%, respectively. This is primarily because when operating on CNG, dual fuel vehicles experience more complete combustion due to the higher-octane rating and cleaner-burning properties of CNG. These results reduced CO and HC emissions in comparison to gasoline vehicles.

The findings also demonstrate that the hybrid vehicle exhibits a notable reduction in NOx emissions compared to conventional vehicles. This reduction is attributed to the use of the electric motor in the hybrid engine, which leads to lower power output and subsequently reduced in cylinder combustion temperature. Elevated temperature is a critical factor in NOx generation, and reducing the temperature leads to a decrease in NOx production.

*In “****Effect of engine downsizing on fuel consumption and emissions”*** *Section:*

The compression ratio and turbocharging are two key factors that influence the operation and exhaust emissions of internal combustion engines [34]. Engine downsizing, which involves replacing a larger, naturally aspirated engine with a smaller, turbocharged one, can enhance fuel efficiency and reduce emissions. Turbocharging enhances the engine's volumetric efficiency by utilizing waste heat from exhaust gases to power the air compression and pumping mechanism [35]. A comparison of naturally aspirated and turbocharged engines is presented in Fig. 11. The same procedure is repeated for standard driving cycles, as demonstrated in Table 7. Since turbocharger activation is dependent on driving conditions and the demand for extra power, the fuel consumption and CO_2_ emissions of both vehicles exhibit some similarity.

The results of this study demonstrate that engine downsizing, as implemented in vehicle #2, can lead to improved fuel consumption and reduced emissions under various real driving conditions. As shown in Fig. 11, vehicle #2 exhibits lower fuel consumption under real driving cycles, except for sample routes 1 and 4, which demonstrate higher fuel consumption. This finding is consistent with previous research, which has shown that engine downsizing can lead to reduced fuel consumption and emissions [36]. However, it is contingent on other factors such as traffic and speed under real conditions.

In terms of CO_2_ emissions, the findings of this study are consistent with previous research that has demonstrated that reducing the cylinder capacity of a turbocharged engine can lead to decreased specific emissions of CO_2_ [35, 37]. As depicted in Fig. 11, the CO_2_ emissions of the naturally aspirated engine exceeded those of the turbocharged engine in all driving cycles. In contrast, the NOx emissions in the naturally aspirated engine were significantly higher (more than 90%) compared to the turbocharged vehicle [37]. The benefits of turbocharging in reducing NOx emissions are well-established, while simultaneously enhancing power density [38]. This is due to the fact that turbocharging allows for more efficient combustion, thereby reducing the formation of NOx emissions.

In terms of CO and HC emissions, the findings of this study demonstrated that turbocharged engines emit higher levels of CO and HC compared to naturally aspirated engines [39]. This is because turbocharged engines are designed to optimize high-speed performance, providing more power and torque at higher speeds, typical of motorway driving. However, during urban and rural trips, naturally aspirated vehicles emitted lower levels of CO compared to turbocharged vehicles [39]. This is because naturally aspirated engines tend to operate at lower speeds and lower loads, which reduces the formation of CO emissions. In contrast, during motorway trips, turbocharged engines produced higher levels of CO due to the increased load and speed at which they operate. These findings highlight the importance of considering the specific driving conditions and engine operating characteristics when evaluating the effects of engine downsizing on fuel consumption and emissions.

*In “****Effect of body style on fuel consumption and emissions****”* *Section:*

For the purpose of comparing the effects of body style on fuel consumption and emissions, vehicle #3, an SUV, and vehicle #2, a sedan, both equipped with turbocharged engines, were selected. Notably, vehicle #3 is equipped with an automatic transmission, whereas vehicle #2 has a manual transmission. The comparative analysis of these vehicles under real driving cycles is presented in Fig. 12, while the fuel consumption and emissions under standard driving cycles are summarized in Table 8.

It is evident that the sedan vehicle exhibited lower fuel consumption, HC, CO, and CO_2_ emissions compared to the SUV, with a reduction of 13% for fuel consumption, 14% for HC, 20% for CO, and 15% for CO_2_. The disparity in fuel consumption and emissions between the two vehicles can be attributed to several factors [40]. One of the primary factors contributing to this disparity is the weight difference between the two vehicles. SUVs typically have a greater weight than sedans, which results in an increased load on the engine. This increased load is compensated by stronger engine power, leading to higher fuel consumption. Furthermore, SUVs tend to have larger engine volumes and higher volumes of exhaust gases, which also contribute to increased fuel consumption and emissions.

Vehicle #3 investigated in this study, had a weight parameter of 283 kg more than vehicle #2 in the Virtual Vehicle Composer model. This increased weight is expected to contribute to higher fuel consumption and emissions. Additionally, the SUV's design characteristics, such as its taller cabin and larger radius wheels, make it more challenging to optimize aerodynamics. The cabin's increased resistance against wind pressure and the higher drag coefficient of the vehicle contribute to increased fuel consumption and emissions.

Under standard driving cycles, the results show that vehicle #2 exhibited higher levels of HC and CO emissions compared to vehicle #3. The cold start phase of the driving cycles is a crucial factor contributing to these differences. The FTP-75 and UDDS driving cycles involve high speeds during the cold start phase, which can influence driver behavior and gear shift delay. The engine's behavior also changes during this phase, leading to the use of an enriched fuel mixture to prevent incomplete combustion. As a result, higher levels of HC and CO emissions are released during this phase.

*In “****Conclusions and future work****”* *Section:*

The major findings from comparing the driving characteristic parameters, fuel consumption, and emissions of four conventional vehicles and one hybrid vehicle are as follows:

- It was found that driving characteristic parameters exhibit distinct relationships with fuel consumption and emissions under various conditions and vehicle technologies.
- Gasoline and dual fuel vehicles exhibit similar behaviors under traffic conditions, with the exception of slightly higher fuel consumption (approximately 18%) and increased emissions in the gasoline vehicle. In contrast, hybrid vehicle demonstrated an advantage in fuel consumption during idle time, with reductions of around 39% and 26% compared to gasoline and dual fuel vehicles, respectively.
- The turbocharged vehicles yield a notable benefit in terms of fuel consumption and CO_2_ emissions, particularly at high speeds where the turbocharger is activated. In this scenario, the turbocharged vehicle consumes approximately 6% less fuel and 19% less CO_2_ than its naturally aspirated vehicle.
- Regarding specific vehicle technologies, it was observed that hybrid powertrains and the presence of turbochargers had the highest emissions in terms of CO and HC, while exhibiting lower NOx emissions.
- The results indicate that SUV with turbocharging technology exhibit lower fuel consumption at high cruising speeds, although the magnitude of this advantage is relatively higher, about 13% compared to sedans. The turbocharged vehicles exhibit elevated levels of CO and HC emissions in real-world driving cycles compared to standard driving cycles, which are characterized by more aggressive driving patterns.

Future research recommendations include capturing real-world driving behavior by incorporating driving data from multiple individuals, potentially through the use of a chasing method. Additionally, exploring emerging technologies such as fully electric powertrains, evaluating different types of transmissions, and assessing the impact of Exhaust Gas Recirculation (EGR).
