# Supplementary material for: Impact of driving characteristic parameters and vehicle type on fuel consumption and emissions performance over real driving cycles
Source: PLoS One. 2025 Jan 13;20(1):e0317098. doi: 10.1371/journal.pone.0317098 (PMC11729959; doi:10.1371/journal.pone.0317098)
Supplement: Attachment — Submitted filename: Response to Reviewers.docx [file pone.0317098.s002.docx]

**Dear Editor-in-Chief,**

PLOS ONE

We are grateful for the opportunity to revise our paper, " **Impact of driving characteristic parameters and vehicle type on fuel consumption and emissions performance over real driving cycles** " We appreciate the valuable feedback and comments provided by the reviewers and editors, and thank them for their time and effort in reviewing our manuscript. In this revised version, we have thoroughly addressed the reviewer’s comments.

We believe that the changes made in this revision have improved the manuscript's quality and clarity. We have highlighted the changes made in yellow-colored, and we hope that these revisions address any remaining concerns.

Regards,

**M. Azadi, PhD.**

Faculty of Mechanical Engineering, Semnan University, Semnan, Iran

**Reviewer #3:**

1) Comments have been addressed. Thank you. I noticed in the GitHub repository, one of the fields is in ".rar" format. Is that containing code or data? if so, please specify and consider uploading the files in a cloud environment (google drive, microsoft onedrive, or dropbox) and provide the link here in the github repository. This way, everyone will be able to access the files.

**Answer:** *Thank you for your comment on our paper. I appreciate your attention.* The ".rar" file in the GitHub repository contains code and data. To enhance accessibility, we provide a link in the GitHub repository.

# “**Data availability**

All Code and data related to this study are publicly available at: <https://github.com/elmirabagheri/Driving_Characteristic_and_Development> ”

**Reviewer #4:**

1) The data in the manuscript was initiated by TTO several years ago, is it with timeliness? And only one driver was recruited to conduct the experiment, did the driver know the proficiency level of these different types of vehicles?

**Answer:** *Thank you for bringing this to our attention. The data utilized in this study was collected from the Transport and Traffic Organization (TTO) several years ago in Tehran city. While the age of the data may raise concerns about its timeliness, it is important to note that the traffic patterns and routes in urban areas like Tehran often exhibit relative consistency over time, making historical data still relevant for analysis and comparison. Furthermore, the infrastructure and road layouts in the city have remained largely unchanged in recent years, further supporting the validity of using older data for the study.*

“The sample routes in this study were initiated by analyzing data obtained from the Transport and Traffic Organization (TTO) of previous years in Tehran city. Despite the age of the data, the ongoing relevance of the TTO data lies in the consistent traffic patterns and infrastructure layouts of Tehran city, which have remained relatively stable over time. The report provided statistics on major districts, quantifying the proportion of high-volume traffic in different types of routes in Tehran city.”

*In this study, it is important to note that the driver involved was informed to maintain a consistent proficiency level across different vehicle types. By ensuring that there was a same driver in operating all vehicles, we aimed to eliminate potential biases stemming from varying driver proficiencies.*

“The driver, a citizen with extensive driving experience, having approximately 19 years of driving experience, and familiarity with the road conditions in Tehran, was responsible for navigating all routes to minimize the impact of their behavior on the test results [23]. It is important to note that the driver's proficiency was consistent across all vehicles used in the study, ensuring a standardized approach to the assessment of factors affecting fuel consumption and emissions, such as gear shift delays, acceleration from vehicle takeoff, and lane changing patterns.”

2) Figure 3 shows that there is little difference in the percentage of variance of driving characteristics parameters. Please mark the specific percentage values at the top to make it clear.

**Answer:** *Thanks for your thoughtful suggestion, figure 3 now has revised to show percentage values as follow.*

**Fig. 3.** The variance percentage of driving characteristic parameters

3) Please explain in details why choose these 12 driving characteristics and why reduce them to two sensitive parameters, why not 3 or 4···?

**Answer:** *Thank you for your insightful comment. We have chosen these 12 driving characteristic parameters because they are widely recognized in previous research as essential factors. Also, these parameters provide a comprehensive view and encompass almost all relevant driving influences in this context, making them a solid foundation for the analysis.* *Regarding the reduction to two sensitive parameters, this decision was based on a few key considerations. Firstly, dimensionality reduction to two parameters facilitates the visualization and analysis of the data in a two-dimensional space, allowing for clearer interpretation of clustering patterns. While it is possible to reduce the characteristics to three or four parameters, our analysis indicated that the two parameters selected provided a sufficient representation of the variability in the data and captured the most critical relationships, making them more effective for clustering purposes.*

“In the next step, a set of selected driving characteristic parameters, including percentage of driving, idling, cruising, accelerating, decelerating, average speed of trip, average driving speed, maximum speed, maximum negative/positive acceleration, and the standard deviation of acceleration/speed, were employed to describe the attributes of each micro-trip [24]. This comprehensive selection of parameters is grounded in previous research, highlighting their significance in understanding driving behavior, and their relation with fuel consumption and emissions.

Following the extraction of the principal components, the parameters with high variance in these components are identified as sensitive parameters, which are deemed to have a significant impact on the underlying patterns and relationships within the data. To ensure a focused analysis, we reduced the dimensionality to the two parameters that accounted for the highest variance percentages. This approach effectively captures the most critical relationships within the data, making these parameters essential inputs for subsequent modeling and analysis. Fig. 3 illustrates the distribution of variance percentages for the driving characteristic parameters of different vehicles across various sample routes. The first two parameters of each with the highest variance percentage were considered as sensitive parameters.”

4) In the result, the comparisons among driving characteristics parameters, powertrain type, engine miniaturization and body style on fuel consumption and emissions are too repetitive, should be optimized more briefly.

**Answer:** *Thank you for your suggestion. The result section is optimized as follow.*

**“Effect of Driving Characteristics on Fuel Consumption and Emissions**

Driving behavior, traffic conditions, vehicle technology, and sample routes significantly influence a vehicle's driving characteristics, fuel consumption, and emissions. Each vehicle has a unique driving cycle, leading to different parameter values. Table 4 presents driving characteristic parameters, while Table 5 shows fuel consumption and emissions, measured in l/100km, g/km, and g/mi, respectively. The sample routes are identified by R1, R2, R3, and R4, which correspond to routes 1, 2, 3, and 4, respectively.

Fuel consumption and emissions are affected by multiple factors, with key contributors being traffic congestion, which leads to prolonged idling and reduced efficiency, and aggressive driving behaviors like excessive speed and acceleration. The vehicle's technology, including the engine management system and emission controls, also play a crucial role in determining fuel consumption and emissions. To illustrate the effects of these parameters on fuel consumption and emissions, the driving behavior of five vehicles on four sample routes were examined.

Results show that vehicles with less traffic congestion (shorter idling times) have lower fuel consumption and emissions. For instance, vehicle #1 on route 4 had only 0.83% idling time, resulting in lower fuel use and emissions. Extended idling can consume more fuel than restarting; however, restarting increases CO, HC, and NOx emissions [33]. Interestingly, vehicle #4 on route 1 had the highest idling percentage (15.59%) but lower emissions, likely due to low acceleration and an average speed of 43.92 km/h.

Turbocharged vehicle #2 on route 4, with only 1% idling time and a high acceleration rate (44.03%), showed increased CO and HC emissions, despite relatively low fuel consumption and CO_2_ emissions, possibly due to turbocharging at high speeds. For this turbocharged vehicle, an average driving speed tend to exhibit higher emissions of CO and HC, whereas those with lower average driving speeds tend to exhibit lower emissions [18]. Vehicle #3, a turbocharged SUV, displayed this trend as well. On route 1, it faced heavy traffic, resulting in lower average speed (35.03 km/h) and higher fuel consumption and CO_2_ emissions [34].

Other driving characteristic parameters, such as the standard deviation of speed and acceleration, and idling time, correlate significantly with fuel consumption and emissions. Increased idling time and higher standard deviations of acceleration and speed are associated with increased fuel consumption and emissions in conventional vehicles. Conversely, a higher standard deviation of speed corresponds to lower CO and HC emissions in conventional vehicles, while the trend is reversed for turbocharged vehicles.

Vehicle #5 uniquely reduces fuel consumption during idling [35]. On route 3, it had longer idle times and lower average speeds, leading to decreased fuel consumption, CO, and HC emissions, though CO2 emissions were higher.

As NOx pollution is relatively low and comparable across vehicles, it has not been individually assessed for each vehicle. The results indicate that aggressive driving behaviors contribute to increased NOx emissions [36]. Furthermore, frequent instances of stopping the engine while idling have been observed to result in higher NOx emissions.

**Effect of Powertrain Type on Fuel Consumption and Emissions**

To assess the impact of powertrain type on fuel consumption and emissions, a comparative study was conducted on three vehicles: a gasoline vehicle (vehicle #1), a hybrid vehicle (vehicle #5), and a dual fuel vehicle (vehicle #4). Results are illustrated in the Box & Whisker diagram in Fig. 10 and summarized in Table 6.

The hybrid vehicle shows significant advantages, with approximately 39% lower fuel consumption than gasoline and 26% lower than dual fuel vehicles due to efficient energy capture during deceleration [37]. It also exhibits 34% less CO_2_ emissions than vehicle #1 and 10% less than vehicle #4, according to engine shutdown during idling and the use of electric energy sources [37]. Unique subsystems like brushless motors and planetary gearboxes enhance energy efficiency.

Vehicle #4, the dual fuel option, demonstrates an 18% improvement in fuel efficiency compared to gasoline vehicles. Compressed Natural Gas (CNG) has a lower carbon content, resulting in about 28% lower CO_2_ emissions compared to vehicle #1 [38, 39].

In terms of emissions, the hybrid vehicle emits more CO than gasoline and dual fuel vehicles due to increased power demands on the internal combustion engine. Primarily due to the increased power demands placed on internal combustion engines in hybrid electric vehicles, which are necessitated by the simultaneous powering of both wheels and batteries. This may also be attributed to the shorter running time and longer intervals between engine cycles may, which may result in inadequate engine warm-up, thereby contributing to increased CO emissions [15]. In contrast, HC emissions are lower in hybrids under standard cycles but can increase under real conditions. Gasoline vehicles emit 45% more CO and 39% more HC than dual fuel vehicles, owing to the more complete combustion with CNG.

The findings also demonstrate that the hybrid vehicle exhibits a notable reduction in NOx emissions compared to conventional vehicles. This reduction is attributed to the use of the electric motor in the hybrid engine, which leads to lower power output and subsequently reduced in cylinder combustion temperature. Elevated temperature is a critical factor in NOx generation, and reducing the temperature leads to a decrease in NOx production.

**Effect of Engine Downsizing on Fuel Consumption and Emissions**

The compression ratio and turbocharging significantly influence the operation and emissions of internal combustion engines [40]. Engine downsizing, which replaces larger naturally aspirated engines with smaller turbocharged ones, improves fuel efficiency and reduces emissions. Turbocharging enhances volumetric efficiency by using exhaust waste heat for air compression [41]. Fig. 11 compares naturally aspirated and turbocharged engines, while Table 7 presents similar results for standard driving cycles. Turbocharger activation depends on driving conditions, resulting in comparable fuel consumption and CO_2_ emissions between vehicles.

This study shows that engine downsizing in vehicle #2 leads to better fuel efficiency, except for higher consumption in sample routes 1 and 4. This aligns with previous research indicating that downsizing can cut fuel use and emissions [42], although it depends on traffic and speed. The results also confirm that reducing the capacity of turbocharged engines lowers CO_2_ emissions [41, 43]. In all driving cycles, CO_2_ emissions were higher in naturally aspirated engines, while NOx emissions were over 90% greater in naturally aspirated engines compared to turbocharged vehicles [43]. The benefits of turbocharging in reducing NOx emissions are well-established, while simultaneously enhancing power density [44]. This is due to the fact that turbocharging allows for more efficient combustion, thereby reducing the formation of NOx emissions.

For CO and HC emissions, turbocharged engines typically emit more CO and HC than naturally aspirated ones [45]. This is due to their design for high-speed performance. In urban and rural settings, naturally aspirated vehicles produce lower CO emissions because of their operation at lower speeds and loads. Conversely, turbocharged engines generate more CO during motorway driving due to higher loads and speeds. These findings emphasize the need to consider specific driving conditions and engine characteristics when assessing the effects of engine downsizing on fuel consumption and emissions.

**Effect of Body Style on Fuel Consumption and Emissions**

To compare the effects of body style on fuel consumption and emissions, we selected vehicle #3, an SUV, and vehicle #2, a sedan, both equipped with turbocharged engines. Notably, vehicle #3 features an automatic transmission, while vehicle #2 is equipped with a manual transmission. The comparative analysis of these vehicles under real driving cycles is illustrated in Fig. 12, and the summary of their fuel consumption and emissions under standardized driving cycles is presented in Table 8.

It is evident that the sedan vehicle exhibited lower fuel consumption, HC, CO, and CO_2_ emissions compared to the SUV, with a reduction of 13% for fuel consumption, 14% for HC, 20% for CO, and 15% for CO_2_. The disparity in fuel consumption and emissions between the two vehicles can be attributed to several factors [46]. One of the primary factors contributing to this disparity is the weight difference. SUVs typically have a greater weight than sedans, which results in an increased load on the engine. This increased load is compensated by stronger engine power, leading to higher fuel consumption. Furthermore, SUVs tend to have larger engine volumes and higher volumes of exhaust gases, which also contribute to increased fuel consumption and emissions.

Additionally, the design characteristics of SUVs, including a taller cabin and larger wheel radius, hinder aerodynamic optimization. The increased wind resistance and higher drag coefficient associated with SUVs exacerbate fuel consumption and emissions.

Interestingly, under standardized driving cycles, vehicle #2 exhibited elevated levels of HC and CO emissions as compared to vehicle #3. This can largely be attributed to the cold start phase of the driving cycles. During the FTP-75 and UDDS cycles, high speeds are encountered shortly after starting, which can affect driver behavior and lead to gear shift delays. Furthermore, the engine's operation during cold starts often necessitates the use of an enriched fuel mixture to prevent incomplete combustion, subsequently resulting in increased HC and CO emissions during this phase.”

5) A flow chart should be added to show the details on the vehicle simulation steps executed in the MATLAB/Simulink program.

**Answer:** *Thank you for your valuable feedback regarding the inclusion of a flow chart to depict the vehicle simulation steps. In response, I would like to highlight that Figure 6 has been included in the paper specifically to illustrate the vehicle simulation steps executed in the MATLAB/Simulink program. This figure provides a detailed visual representation of the simulation process, outlining each step clearly. We believe that this format effectively conveys the necessary information.*

**Fig. 6.** Steps and structure of designing real vehicle models with MATLAB Virtual Vehicle Composer

**Reviewer #8:**

The manuscript proposes "Impact of driving characteristic parameters and vehicle type on fuel consumption and emissions performance over real driving cycles." The article shows a lack of innovation, detailed comments are as follows,

1) Is it too little data based on 4 traditional vehicles and 1 hybrid vehicle, and can it reflect driving characteristics? Are the drivers of the 5 cars the same? Is the driver consistent for each driving cycle? The driving style is only reflected in speed and acceleration, which is very one-sided. In addition, vehicle color, as a parameter, is also a variable, but it can only have 5 values. The same problem applies to other parameters, and limited data cannot lead to convincing conclusions.

**Answer:** *Thank you for your insightful comments. There are some considerations that should be mentioned. The choice of vehicles and the ratio of conventional to hybrid (i.e. 4 to 1) is based on the conditions of the studied area. Each vehicle represents a wide range of vehicle platforms available in Tehran city, which have very similar specifications in terms of powertrain, engine downsizing, and body style. In addition, for each of the five vehicles, different micro-trips have been considered, each of which alone has 12 driving characteristics, including percentage of driving, idling, cruising, accelerating, decelerating, average driving speed, average speed of trip, maximum speed, maximum negative/positive acceleration, and the standard deviation of acceleration/speed.*

*The driver of all vehicles has been the same in all routes, which provides a condition for a fair comparison. Also, all the parameters selected in this study are influential factors in modeling that have been mentioned in previous researches in the literature. Other side factors, such as the color of the vehicles, have a very small effect on the results (fuel consumption and emissions), so they are ignored. In general, the most famous drive cycles in transportation, among which we can mention UDDS and FTP-75, consist of two main components "speed" and "acceleration" and its derivatives. All the drive cycles presented in this article are made according to the existing drive cycle standards.*

“This comprehensive selection of parameters is grounded in previous research, highlighting their significance in understanding driving behavior, and their relation with fuel consumption and emissions.”

2) The theoretical innovation of the article is not obvious, and it mentions real driving cycles, which were simulated using MATLAB. How to integrate real and simulated data? Moreover, in the simulation, there is no detailed introduction on how to distinguish between traditional vehicles and hybrid vehicles, and how to set the simulation environment and parameters.

**Answer:** *Thank you for your comments. In general, the theoretical innovations of the article can be considered in two parts i) real drive cycles and ii) vehicle simulation data in MATLAB. To address the integration of real and simulated data, we employed real driving cycles generated through the K-means and PCA algorithms, which reflect actual driving behavior in Tehran’s unique traffic conditions. These real cycles are considered as input data for vehicle simulation in MATLAB/Simulink, allowing us to simulate how different vehicle types respond to varying driving conditions accurately. This issue has been clarified in the revised manuscript.*

*As stated in the revised version, the simulated model of each car consists of six blocks as shown in Figure 6. The parameter values for the vehicles are detailed in Table 3 and Figure 8. Also, the hybrid car has a separate block from conventional vehicles, whose structure is drawn in Figure 9. In line with your opinion, the complete details related to the simulation of conventional and hybrid vehicles in Simulink and the parameters of each, have been uploaded in the GitHub link of the article.*

“In this section, vehicles are simulated by using MATLAB/Simulink with the Powertrain Blockset, which is a powerful tool for data-driven modeling and analyzing the performance of vehicles [30]. The dynamic modeling of five vehicles and its components is simulated in accordance with the characteristics of each vehicle in this Blockset. In the following, the obtained drive cycles are considered as simulation input and are fed to the model.”

3) If all 12 driving characteristic parameters are related to speed and acceleration, then in each driving cycle, if the driver is the same, this style will be severely correlated with the driver's driving style, which will lead to significant unreliability in the result analysis.

**Answer:** *Thank you for your insightful comment regarding the potential influence of driving style on the analysis of driving characteristic parameters. To address this concern, we ensured that the same driver operated all vehicles across various driving cycles. This approach was taken specifically to minimize variability introduced by different driving styles and to isolate the vehicle characteristics being studied. By maintaining a consistent driving pattern, we aimed to create a controlled environment that allows for a more reliable comparison of vehicle performance related to engine, powertrain configurations, and etc. Recognizing that driver behavior can significantly impact driving characteristics, we believe that the methodology employed effectively mitigates these effects. The focus was not solely on the individual driver’s behavior but rather on how each vehicle's design and technology such as engine size, turbocharging, and powertrain type affect the overall fuel consumption and emissions outcomes. Despite the inherent correlation between driving style and speed/acceleration metrics, the consistent driving conditions help ensure that any differences observed in fuel consumption and emissions can be more confidently attributed to the vehicles themselves rather than the driver's idiosyncratic behavior.*

“The driver, a citizen with extensive driving experience, having approximately 19 years of driving experience, and familiarity with the road conditions in Tehran, was responsible for navigating all routes to minimize the impact of their behavior on the test results [23]. It is important to note that the driver's proficiency was consistent across all vehicles used in the study, ensuring a standardized approach to the assessment of factors affecting fuel consumption and emissions, such as gear shift delays, acceleration from vehicle takeoff, and lane changing patterns. This approach allows for a more reliable analysis of vehicle characteristics, ensuring that any observed differences in fuel consumption and emissions can be attributed primarily to the vehicle designs and technologies rather than the driver’s behavior.”

**Reviewer #9:**

1) Data Collection Frequency: The data collection frequency is set at 1 Hz. How does this frequency impact the accuracy of the driving characteristic parameters, especially during high-speed maneuvers?

**Answer:** *Thank you for your valuable comment regarding the data collection frequency set at 1 Hz and its potential impact on the accuracy of driving characteristic parameters during high-speed maneuvers. In our research, the decision to utilize a 1 Hz data collection frequency was made with careful consideration of the trade-offs between data granularity and the practicality of data handling. At this frequency, speed, position, and other parameters are recorded once per second, which is generally sufficient for capturing typical driving behaviors and conditions in urban and highway settings.*

“In this research, speed data from vehicles was collected using a GPS logger software, which tracked positions and routes, enabling the monitoring of trips and the retrieval of location, speed, altitude, direction, and various statistics. The software allows for the review of recorded trips at any time and provides output in GPX, KML, and TXT formats. The data collection frequency was set at 1 Hz, a decision made with careful consideration of the trade-offs between data granularity and practicality in handling data. The software was installed on a Samsung Galaxy S21 mobile phone.”

2) Clustering Technique Justification: The authors employed the K-means algorithm for clustering micro-trips. What specific characteristics of the data justified the choice of K-means over other clustering methods?

**Answer:** *Thank you for your insightful comment. In our analysis, we conducted a thorough evaluation of the characteristics of the data and the objectives of our clustering approach. The K-means algorithm was selected for several key reasons:*

1. *Simplicity and Interpretability: K-means is known for its straightforward implementation and ease of interpretation. Given that our goal was to categorize micro-trips based on specific driving conditions, the transparent nature of K-means allows for clearer insights into how clusters are formed.*
2. *Scalability: With the volume of data we collected, K-means demonstrates strong performance in terms of computational efficiency. The algorithm can handle large datasets effectively, making it suitable for our micro-trip analysis where we aimed to process numerous driving records.*
3. *Distance-Based Clustering: K-means operates on the principle of minimizing intra-cluster variance, making it particularly effective for our application, which focuses on grouping micro-trips that exhibit similar driving behaviors (e.g., speed patterns). This distance-based approach aligns well with our objective of identifying distinct driving patterns in the data.*

*In summary, the choice of K-means was driven by its efficiency, simplicity, and suitability for our specific data characteristics and research objectives.*

“The K-means algorithm is employed as the clustering technique, which is widely used in similar domains due to its efficiency, simplicity, and suitability particularly when dealing with large datasets [27]. The choice of K-means is justified based on several characteristics of the data and the specific objectives of the clustering approach. The simplicity and interpretability of K-means provide a clear framework for categorizing micro-trips based on distinct driving conditions. Additionally, K-means demonstrates strong scalability, allowing for efficient processing of significant volumes of driving records without compromising performance. The algorithm's distance-based clustering approach, which minimizes intra-cluster variance, is particularly effective for grouping micro-trips that exhibit similar driving behaviors, such as speed patterns. Overall, these factors contribute to K-means being the most appropriate clustering technique for this study.”

3) Parameter Selection: The study identifies 12 driving characteristic parameters. What criteria were used to select these parameters, and how do they correlate with fuel consumption and emissions?

**Answer:** *Thank you for your thoughtful comment. The selection of the driving characteristic parameters in our study was guided by several criteria that align with established research and practice in the field of transportation and environmental impact analysis:*

*1. Relevance to Driving Behavior: The parameters were chosen based on their direct influence on vehicle operation and efficiency. 2. Relationship with Fuel Consumption and Emissions: Each of the selected parameters has been identified in prior studies as being correlated with fuel efficiency and greenhouse gas emissions. And 3. Diversity of Parameter.*

“In the next step, a set of selected driving characteristic parameters, including percentage of driving, idling, cruising, accelerating, decelerating, average driving speed, average speed of trip, maximum speed, maximum negative/positive acceleration, and the standard deviation of acceleration/speed, were employed to describe the attributes of each micro-trip [24]. This comprehensive selection of parameters is grounded in previous research, highlighting their significance in understanding driving behavior, and their relation with fuel consumption and emissions.”

4) Impact of Weather Conditions: While data was collected under sunny conditions, how might variations in weather (e.g., rain, snow) affect the driving cycles and the resulting fuel consumption and emissions?

**Answer:** *Thanks for insightful comment. While our data collection focused on sunny weather conditions to ensure consistency and eliminate the confounding effects of adverse conditions, it is important to acknowledge that variations in weather can significantly influence driving behavior and vehicle performance. For example: Adverse weather conditions, such as rain or snow, often lead drivers to alter their driving patterns. For example, drivers may reduce speed and increase following distances to maintain safety, which could result in lower acceleration rates but potentially longer travel times. Also, rain, snow, and icy conditions can create additional resistance for vehicles. Wet roads can increase drag, requiring more energy (and thus more fuel) to maintain speed. Snow and ice can further exacerbate this condition. In addition, weather conditions, particularly cold temperatures or heavy rain, may increase the use of vehicle auxiliary systems, such as heating, air conditioning, and windshield wipers. These systems can draw additional power, thereby impacting fuel efficiency and increasing emissions.*

“Data collection was conducted in February, June, and July 2022, specifically under sunny weather conditions to ensure consistency and eliminate the confounding effects of adverse weather. While this approach helps maintain reliable results, it is important to acknowledge that variations in weather can significantly influence driving behavior and vehicle performance. Adverse weather, such as rain or snow, often leads to changes in driving patterns. Additionally, wet roads can increase drag, requiring more energy (and thus more fuel) to maintain speed. Snow and ice can exacerbate these effects. Furthermore, adverse conditions, particularly cold temperatures or heavy rain, can necessitate greater use of vehicle auxiliary systems like heating, air conditioning, and windshield wipers, which draw additional power, impacting fuel efficiency and increasing emissions.”

5) Sample Size and Representativeness: The study examines five vehicles. Is this sample size sufficient to draw generalizable conclusions about vehicle performance across different models and brands?

**Answer:** *Thank you for your thoughtful comment. While a sample size of five vehicles may appear limited, due to the cost and time. However, the selected vehicles encompass a range of models and brands, capturing variations in engine technology, fuel types, and design philosophies. This diversity is crucial for analyzing performance and emissions under similar driving conditions.*

6) Future Research Directions: The authors mention the need for further research. What specific areas do they suggest for future studies to build upon their findings?

**Answer:** *Thank you for your feedback. In our paper, we will suggest several specific areas such as high-traffic environments in metropolitan areas, as these locations present unique challenges and behaviors that may influence vehicle performance, emissions, and efficiency.*

“Future research recommendations include capturing real-world driving behavior by incorporating driving data from multiple individuals, potentially through the use of a chasing method, specially in high traffic areas such as metropolitan. Additionally, exploring emerging technologies such as fully electric powertrains, evaluating different types of transmissions, and assessing the impact of Exhaust Gas Recirculation (EGR).”

7) Practical Implications: What practical implications do the authors foresee from their findings for policymakers and automotive manufacturers in terms of improving vehicle technologies and urban planning?

**Answer:** *Thank you for the thoughtful comment. The findings of this research offer valuable insights for decision-making in vehicle technology and urban planning:*

*In summary, the research findings provide actionable insights for policymakers and automotive manufacturers, emphasizing the importance of refining emission standards, promoting hybrid and electric vehicles, and enhancing urban traffic management. By fostering collaboration among stakeholders and implementing targeted incentives and educational initiatives, a significant impact can be made on reducing emissions and shaping sustainable urban environments.*

“Ultimately, these findings not only underscore the need for refined policies and regulations based on real-world data but also highlight the potential for innovative vehicle technologies and infrastructure improvements to facilitate the transition to greener alternatives in urban transportation.” (END OF CONCLUSION)
