# Supplementary material for: Impact of driving characteristic parameters and vehicle type on fuel consumption and emissions performance over real driving cycles
Source: PLoS One. 2025 Jan 13;20(1):e0317098. doi: 10.1371/journal.pone.0317098 (PMC11729959; doi:10.1371/journal.pone.0317098)
Supplement: Attachment — Submitted filename: Response to Editor.docx [file pone.0317098.s003.docx]

**Dear Editor-in-Chief,**

PLOS ONE

We sincerely appreciate the opportunity to revise our paper, " **Impact of driving characteristic parameters and vehicle type on fuel consumption and emissions performance over real driving cycles** " We are thankful for the valuable feedback and comments provided by the reviewers and editors, and thank them for their time and effort in reviewing our manuscript. In this revised edition, we have carefully addressed the comment from the editor.

We believe that the changes made in this revision have improved the manuscript's quality and clarity. We have highlighted the changes made in yellow-colored, and we hope that this revision addresses any remaining concerns.

Regards,

**M. Azadi, PhD.**

Faculty of Mechanical Engineering, Semnan University, Semnan, Iran

**Editor comment:**

1) The inclusion of naturalistic driving in this study is a well-justified and appropriate approach, as it aligns closely with the need to capture real-world driving behaviors that significantly influence fuel consumption and emission levels. Naturalistic driving allows for the extraction of authentic driving patterns, which can then be correlated with specific vehicle performance metrics, providing a comprehensive understanding of the variability introduced by actual road conditions and driving styles. To strengthen the impact of this work, it is recommended to incorporate references to relevant studies that have successfully utilized methodologies such as Geographic Information Systems (GIS) or Global Navigation Satellite Systems (GNSS) to extract and analyze driving patterns. These studies demonstrate the critical role of spatial and kinematic data in representing driving behavior and its subsequent relationship to emissions and fuel efficiency (Extraction of naturalistic driving patterns or Representation of kinematic driving behaviour). Highlighting these methodologies can further underscore the robustness of the current approach while situating the work within the broader scientific context.

**Answer:** *Thank you for your insightful feedback regarding the inclusion of naturalistic driving in my study. I appreciate your suggestion to enhance the manuscript by referencing relevant methodologies, particularly the use of Geographic Information Systems (GIS) and Global Navigation Satellite Systems (GNSS). In response, we have incorporated a discussion of recent advancements in these methodologies, emphasizing how GIS has been utilized as mentioned in the below. I believe these additions will strengthen the overall impact of the paper and situate it within the broader scientific context. Thank you once again for your valuable recommendations.*

Typically, driving cycles are employed to evaluate the performance of vehicles, including emission rates and fuel consumption [5], as well as in vehicle simulations [6]. In recent years, numerous studies have been conducted to decrease fuel consumption and emissions. These studies can be analyzed from different perspectives and can be categorized according to their common features, including cleaner internal combustion engines, hybrid or electric vehicles [7], engine downsizing, alternative fuels, and improving the road network topography [8]. However, one crucial and often overlooked factor is the behavior of the driver, which significantly impacts a vehicle's fuel consumption and emissions. Researchers have observed that driving habits and the energy efficiency of vehicles are influenced by traffic conditions [9, 10]. Their findings suggest that in congested areas, average fuel consumption tends to increase, regardless of the specific road section [11]. Furthermore, studying changes in driving characteristic parameters, such as instantaneous acceleration and speed, is essential because they have a significant effect on exhaust emissions and fuel consumption [12]. Recent advancements in methodologies, particularly the application of Geographic Information Systems (GIS), have provided valuable insights into the complexities of driving behavior [13]. For instance, several studies have implemented GIS to georeference naturalistic driving data, enhancing data representation and visualization, thus enabling researchers to better understand driving patterns [14]. Moreover, innovative frameworks utilizing GIS allow for the extraction and analysis of driving behaviors across various contexts and spatial scales, ultimately contributing to a more comprehensive evaluation of how driver behaviors influence performance metrics such as fuel consumption and emissions [15].

2) Please note that your Data Availability Statement is currently missing. The link you provided is not working. If your manuscript is accepted for publication, you will be asked to provide these details on a very short timeline. We therefore suggest that you provide this information now, though we will not hold up the peer review process if you are unable.

**Answer:** *Thank you for your insightful feedback. We have changed the link, as follows,*

All codes related to this study (for the vehicle simulation with Simulink and for the driving cycle with Matlab) are publicly available at: <https://data.mendeley.com/datasets/f7c75syg2f/1> (Azadi, Mohammad; Bagheri, Elmira; Masih-Tehrani, Masoud; Moosavian, Ashkan (2024), “Codes for the vehicle simulation with Simulink and for the driving cycle with Matlab”, Mendeley Data, V1, doi: 10.17632/f7c75syg2f.1).
